# Comparison of SARS-CoV-2 Hyperimmune Immunoglobulins Following Infection Plus Vaccination vs Infection

**DOI:** 10.1001/jamanetworkopen.2023.27307

**Published:** 2023-08-04

**Authors:** Lorenza Bellusci, Hana Golding, Surender Khurana

**Affiliations:** 1 Division of Viral Products, Center for Biologics Evaluation and Research, Food and Drug Administration, Silver Spring, Maryland

## Abstract

This cross-sectional study compares the neutralizing titers of convalescent plasma and hyperimmune anti–SARS-CoV-2 intravenous immunoglobulins against circulating Omicron subvariants.

## Introduction

Monoclonal antibodies (MAbs), convalescent plasma (CP), and hyperimmune intravenous immunoglobulins (IVIGs) have been used to treat patients with COVID-19, especially immunocompromised patients, who are at high risk for severe disease following SARS-CoV-2 infection.^1,2^ However, newly circulating, highly contagious sublineages of the Omicron variant contain multiple variations in the spike protein, resulting in resistance to therapeutic MAbs as well as antibodies generated by SARS-CoV-2 vaccines (using mRNA technology) or prior SARS-CoV-2 infection.^3^

Recently, survival benefit was observed for patients with COVID-19 and immunodeficiency treated with hyperimmune anti-SARS-CoV-2 IVIGs (hCoV-2IG).^4^ The postinfection hCoV-2IG (pi-hCoV-2IG) lots manufactured from pooled plasma units of convalescent individuals contain immunoglobin G at 10-fold higher concentration than in CP. However, the pi-hCoV-2IG lots prepared from CP collected in 2020 and 2021 plasma donors prior to circulation of Omicron variants demonstrated limited neutralizing antibody titers against previous Omicron variants BA.1 to BA.5.^5^ It was postulated that hCoV-2IG lots prepared from SARS-CoV-2–vaccinated individuals with or without prior infections (Vx-hCoV-2IG) may provide broader protection against circulating Omicron subvariants.

## Methods

This cross-sectional study evaluated the therapeutic potential of pi-hCoV-2IG and Vx-hCoV-2IG against recent Omicron variants (eTable in Supplement 1). We followed the STROBE reporting guideline. This study was approved by the Food and Drug Administration’s Research Involving Human Subjects Committee, and informed consent was not required because we only obtained deidentified, leftover samples from clinicians.

We tested 19 lots of pi-hCoV-2IG prepared from pooled plasma of convalescent individuals infected with SARS-CoV-2 in 2020 and 1 available Vx-hCoV-2IG lot manufactured from pooled plasma of SARS-CoV-2–vaccinated individuals (hybrid immunity) who reported prior COVID-19 infection in 2021 (during the circulation of Alpha and Delta variants). Additionally, 20 IVIG preparations manufactured in 2019 from healthy plasma donations (2019-IVIG) before the COVID-19 pandemic, 8 IVIG lots manufactured in 2020 (2020-IVIG), 8 CP from recovered COVID-19 patients in early 2020 (2020-CP), and 8 CP from Omicron vaccine breakthrough infections in 2022 (2022-CP) (all collected approximately 30 days after diagnosis) were analyzed for neutralization of SARS-CoV-2 ancestral WA-1 and currently circulating Omicron BQ.1, BQ.1.1, XBB.1, and XBB.1.5 subvariants in a pseudovirus neutralization assay (eAppendix in Supplement 1). Pairwise comparisons were analyzed using ordinary 1-way analysis of variance with Tukey pairwise multiple comparison test. The differences were considered statistically significant with a 95% CI when 2-sided *P* < .05. Statistical analysis was performed using GraphPad Prism version 9.3.1 (Dotmatics) from May to June 2023.

## Results

SARS-CoV-2 neutralization titers were determined for 20 prepandemic 2019-IVIG preparations, 8 2020-IVIG preparations, 8 2020-CP preparations, 8 2022-CP preparations, 19 pi-hCoV-2IG preparations, and 1 Vx-hCoV-2IG preparation. The 2019-IVIG preparations manufactured from healthy individuals were negative against SARS-CoV-2. Similarly, only 3 of 8 preparations of 2020-IVIG had low neutralization titers against WA-1 (geometric mean titer [GMT] ranging from 44 to 101) and no neutralization of Omicron subvariants (Figure and Table).

**Figure.**
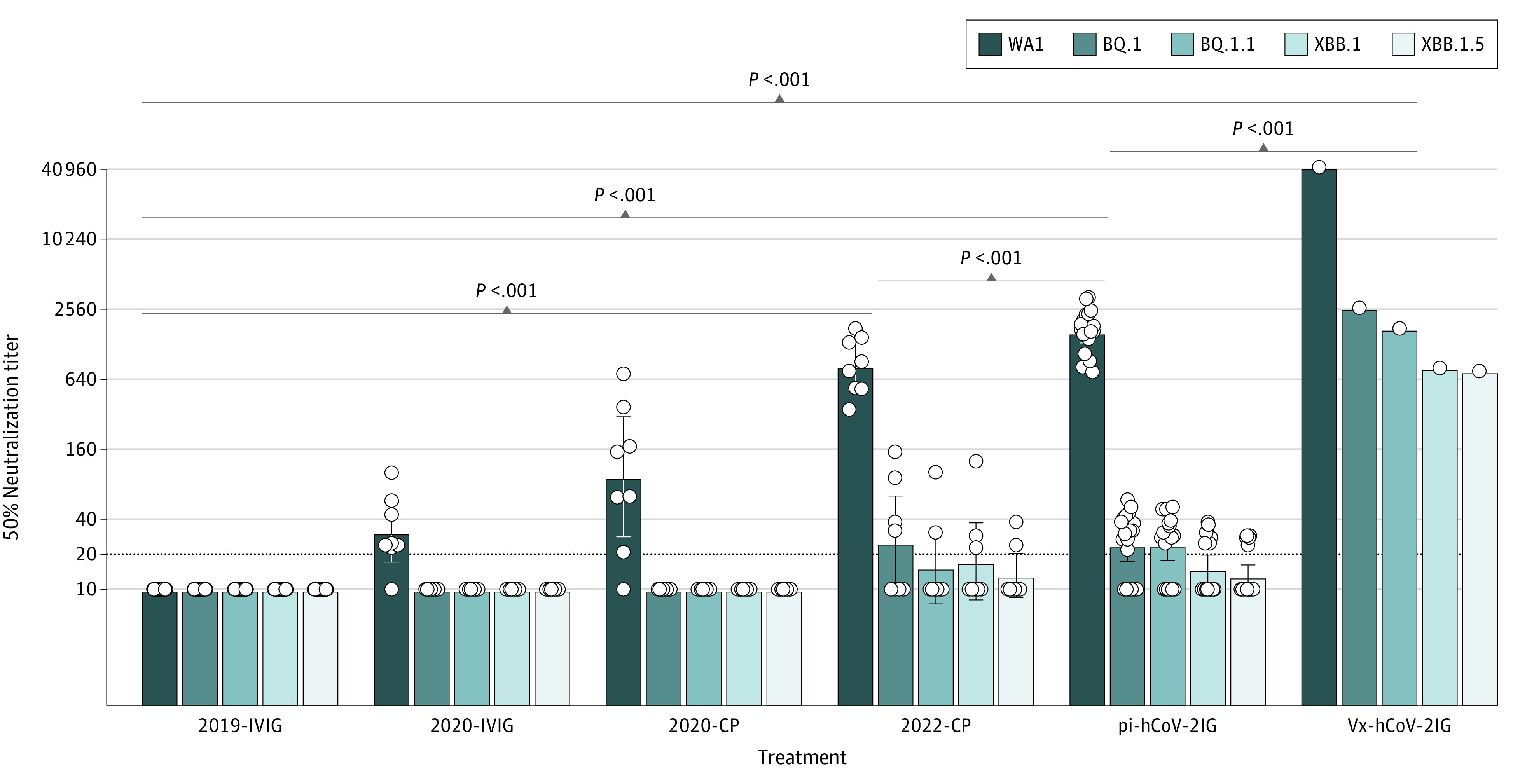
Neutralization of SARS-CoV-2 WA-1/2020 and Circulating Omicron Subvariants by Intravenous Immunoglobulins (IVIG), Convalescent Plasma (CP), Postinfection Hyperimmune Anti–SARS-CoV-2 IVIGs (pi-hCoV-2IG), and SARS-CoV-2–Vaccinated Individuals With or Without Prior Infections (Vx-hCoV-2IG) SARS-CoV-2 neutralization assays were performed by using pseudoviruses expressing the spike protein of WA-1/2020 or the Omicron subvariants in 293-ACE2-TMPRSS2 cells. SARS-CoV-2 neutralization titers were determined in the 2019-IVIG (prepandemic), 2020-CP, 2022-CP, pi-hCoV-2IG, and Vx-hCoV-2IG preparations. Each individual sample titer is shown as a circle. The assay was performed in duplicate to determine the 50% neutralization titer. The heights of the bars indicate the geometric mean titers, and the whiskers indicate 95% CIs. The horizontal dotted line indicates the limit of detection for the neutralization assay (50% neutralization titer of 20). Differences between SARS-CoV-2 strains were analyzed by ordinary 1-way analysis of variance using Tukey pairwise multiple comparison test in GraphPad Prism version 9.3.1 (Dotmatics), and the *P* values are shown.

**Table.** Neutralization Titers of Convalescent Plasma, IVIG, and hCoV-2IG Against SARS-CoV-2 Variants^a^

Lot	SARS-CoV-2 variants, 50% neutralization titer				
	WA-1	BQ.1	BQ.1.1	XBB.1	XBB.1.5
**IVIG lots produced in 2019 prior to COVID-19**					
2019-IVIG-1	10	10	10	10	10
2019-IVIG-2	10	10	10	10	10
2019-IVIG-3	10	10	10	10	10
2019-IVIG-4	10	10	10	10	10
2019-IVIG-5	10	10	10	10	10
2019-IVIG-6	10	10	10	10	10
2019-IVIG-7	10	10	10	10	10
2019-IVIG-8	10	10	10	10	10
2019-IVIG-9	10	10	10	10	10
2019-IVIG-10	10	10	10	10	10
2019-IVIG-11	10	10	10	10	10
2019-IVIG-12	10	10	10	10	10
2019-IVIG-13	10	10	10	10	10
2019-IVIG-14	10	10	10	10	10
2019-IVIG-15	10	10	10	10	10
2019-IVIG-16	10	10	10	10	10
2019-IVIG-17	10	10	10	10	10
2019-IVIG-18	10	10	10	10	10
2019-IVIG-19	10	10	10	10	10
2019-IVIG-20	10	10	10	10	10
**IVIG lots produced in 2020 (circulating SARS-CoV-2 strains: Wuhan, D614G, and Alpha)**					
2020-IVIG-1	23	10	10	10	10
2020-IVIG-2	24	10	10	10	10
2020-IVIG-3	101	10	10	10	10
2020-IVIG-4	25	10	10	10	10
2020-IVIG-5	58	10	10	10	10
2020-IVIG-6	44	10	10	10	10
2020-IVIG-7	24	10	10	10	10
2020-IVIG-8	10	10	10	10	10
**Convalescent plasma lots produced from COVID-19 survivors collected in 2020 (circulating SARS-CoV-2 strains: Wuhan, D614G, and Alpha)**					
2020-CP-1	369	10	10	10	10
2020-CP-2	170	10	10	10	10
2020-CP-3	10	10	10	10	10
2020-CP-4	152	10	10	10	10
2020-CP-5	21	10	10	10	10
2020-CP-6	62	10	10	10	10
2020-CP-7	63	10	10	10	10
2020-CP-8	714	10	10	10	10
**Convalescent plasma lots produced from COVID-19 survivors collected in 2022 (circulating SARS-CoV-2 strains: Omicron BA.1 and BA.2)**					
2022-CP-1	1753	153	102	126	38
2022-CP-2	905	38	10	10	10
2022-CP-3	352	10	10	10	10
2022-CP-4	539	10	10	10	10
2022-CP-5	755	10	10	10	10
2022-CP-6	1331	32	10	10	10
2022-CP-7	1460	91	31	29	24
2022-CP-8	528	10	10	23	10
**pi-hCoV-2IG lots produced from COVID-19 CP donors (circulating SARS-CoV-2 strains: Wuhan, D614G, and Alpha)**					
hCoV-2IG-1	2067	59	49	31	28
hCoV-2IG-2	1664	10	10	10	10
hCoV-2IG-3	1602	37	33	28	24
hCoV-2IG-4	1833	10	10	10	10
hCoV-2IG-5	2302	10	10	10	10
hCoV-2IG-6	920	10	10	10	10
hCoV-2IG-7	3243	45	49	38	29
hCoV-2IG-8	1730	41	28	10	10
hCoV-2IG-9	819	32	25	10	10
hCoV-2IG-10	1428	27	29	10	10
hCoV-2IG-11	737	22	28	10	10
hCoV-2IG-12	917	27	31	10	10
hCoV-2IG-13	1055	10	10	10	10
hCoV-2IG-14	1896	32	35	25	10
hCoV-2IG-15	1565	10	10	10	10
hCoV-2IG-16	2351	30	49	31	28
hCoV-2IG-17	2486	43	37	25	10
hCoV-2IG-18	3162	51	51	36	29
hCoV-2IG-19	1646	38	39	10	10
**Vx-hCoV-2IG lots produced from SARS-CoV-2–vaccinated plasma donors** ^b^ ** (with prior COVID-19 with either Alpha or Delta variants)**					
Vx-hCoV-2IG	42 706	2629	1754	804	755

Abbreviations: CP, convalescent plasma; hCoV-2IG, hyperimmune anti-SARS-CoV2 IVIGs; IVIG, intravenous immunoglobulins; pi-hCoV-2IG, postinfection hCoV-2IG; Vx-hCoV-2IG, SARS-CoV-2–vaccinated individuals with or without prior infections.

^a^ Pseudovirus neutralization assay titer cutoff value was 1:10.

^b^ US plasma donors who received SARS-CoV-2 vaccine with mRNA technology.

The 2020-CP showed variable pseudovirus 50% neutralization assay (PsVNA50) titers against WA-1 ranging between 10 and 714 (GMT, 93) but did not neutralize Omicron variants. In contrast, 2022-CP demonstrated robust PsVNA50 titers against WA-1 (GMT, 834). However, only a few 2022-CP showed neutralization of BQ.1 (GMT, 25), BQ.1.1 (GMT, 15), XBB.1 (GMT, 17), and XBB.1.5 (GMT, 13) (Figure and Table).

The 19 postinfection pi-hCoV-2IG lots demonstrated robust neutralization of WA-1 (GMT, 1615). However, the neutralization titers were very low (or negative) against BQ.1 (GMT, 24), BQ.1.1 (GMT, 24), XBB.1 (GMT, 15) and XBB.1.5 (GMT, 13) variants. The Vx-hCoV-2IG from vaccinated individuals demonstrated approximately 10-fold higher neutralization titer against the ancestral WA-1 strain (GMT, 42 705), as well as high neutralizing antibody titers against BQ.1 (GMT, 2629), BQ.1.1 (GMT, 1754), XBB.1 (GMT, 804), and XBB.1.5 (GMT, 755) variants (Figure and Table). These neutralization titers (PsVNA50 titer >1:40) against the circulating Omicron subvariants are expected to provide protection against severe COVID-19.^6^

## Discussion

This study found that antibodies generated by hybrid immunity following vaccination of adults previously infected with SARS-CoV-2 are more cross-reactive and can neutralize circulating Omicron subvariants, which are resistant to most available therapeutic Mabs, CP, and pi-hCoV-2IG. A limitation of the study is the availability of only 1 postvaccination Vx-hCoV-2-IG lot. However, the data presented should encourage production of new Vx-hCoV-2-IG lots for preexposure or postexposure prophylaxis against severe COVID-19 caused by SARS-CoV-2 emerging variants.

## Notes

Supplement 1. **eAppendix.** Materials and Methods
eReferences
**eTable.** SARS-CoV-2 Variants Mutations Introduced in the Spike Plasmid for Production of SARS-CoV-2 Pseudovirions for Analysis in PsVNA

Supplement 2.
Data Sharing Statement
