# Supplementary material for: Comparison of SARS-CoV-2 Hyperimmune Immunoglobulins Following Infection Plus Vaccination vs Infection
Source: JAMA Netw Open. 2023 Aug 4;6(8):e2327307. doi: 10.1001/jamanetworkopen.2023.27307 (PMC10403779; doi:10.1001/jamanetworkopen.2023.27307)
Supplement: Supplement 1. — eAppendix. Materials and Methods eReferences eTable. SARS-CoV-2 Variants Mutations Introduced in the Spike Plasmid for Production of SARS-CoV-2 Pseudovirions for Analysis in PsVNA [file jamanetwopen-e2327307-s001.pdf]

Supplemental Online Content

Bellusci L, Golding H, Khurana S. Comparison of SARS-CoV-2 hyperimmune immunoglobulins following infection plus vaccination vs infection. *JAMA Netw Open.* 2023;6(8):e2327307. doi:10.1001/jamanetworkopen.2023.27307

eAppendix. Materials and Methods

eReferences

eTable. SARS-CoV-2 Variants Mutations Introduced in the Spike Plasmid for Production of SARS-CoV-2 Pseudovirions for Analysis in PsVNA

This supplemental material has been provided by the authors to give readers additional information about their work.

© 2023 Bellusci L et al. *JAMA Network Open.*

eAppendix. MATERIALS AND METHODS

Samples and study design

IVIG products approved in the United States are polyclonal antibody preparations made from 10,000 or more U.S. plasma donors and may include cold alcohol fractionation (Cohn-Oncley), anion-exchange and size-exclusion chromatography's. The final product is sterile-filtered IgG (>95%) and formulated at 100 mg/mL. Twenty intravenous immunoglobulin batches were produced from plasma collected prior to August 2019 (2019-IVIG) and eight IVIG lots made from plasma donations in 2020 (2020-IVIG) and manufactured between October 2020 and January 2021 (each lot derived from >10,000 donors), were obtained from six manufacturers.

Nineteen pi-hCoV-2IG batches prepared from post-SARS-CoV-2 infection CP collected at least 30-days post-recovery (~200-1000 US plasma donors per lot) were obtained/purchased from four commercial companies for blinded antibody analysis. The plasma units used in the manufacturing of the hCoV-2IG batches were collected in 2020 (during circulation of ancestral Wuhan, D614G or alpha strains) prior to emergence of the Delta and Omicron VOCs or prior to availability of COVID-19 vaccines. One hyperimmune intravenous immunoglobulin lot (Vx-hCoV-2IG) produced from plasma collected from SARS-CoV-2 vaccinated individuals at least 2 weeks after second vaccination (most with prior COVID-19 infection) in 2021 (during the alpha and delta circulation) prior to circulation of Omicron was obtained from one manufacturer.

Eight random CP lots were obtained from recovered COVID-19 patients between May-September 2020 (at least 30-days post-recovery) prior to COVID-19 vaccinations. At the time of collection SARS-CoV-2 D614G strain was the predominant strain in the US. Eight CP lots were collected in February 2022 from recovered individuals (~30 days post-diagnosis) following

© 2023 Bellusci L et al. *JAMA Network Open.*

Omicron breakthrough infections (probably BA.1), who received at least two doses of COVID-mRNA vaccination.

Ethics Statement

This study was approved by the Food and Drug Administration’s Research Involving Human Subjects Committee (RIHSC #2020-04-02). This study complied with all relevant ethical regulations for work with human participants, and informed consent was obtained. Samples were collected from adult subjects who provided informed consent to participate in the study. All assays performed fell within the permissible usages in the original informed consent.

Neutralization assay

Samples were evaluated in a qualified SARS-CoV-2 pseudovirion neutralization assay (PsVNA) using SARS-CoV-2 WA1/2020 strain and circulating Omicron subvariants: BQ.1, BQ.1.1, XBB.1 and XBB.1.5. The mutations in spike protein of these Omicron subvariants are shown in Supplementary Table S1. SARS-CoV-2 neutralizing activity measured by PsVNA correlates with PRNT (plaque reduction neutralization test with authentic SARS-CoV-2 virus) in previous studies (1-3). However, some antibodies targeting the N-terminal domain of SARS-Cov-2 spike may not show neutralization in the pseudovirus neutralization assay.

Neutralization assays were performed as previously described (2, 4). Briefly, 50 µL of SARS-CoV-2 S pseudovirions (counting ~200,000 relative light units) were pre-incubated with an equal volume of medium containing serial dilutions (starting at 1:10) of all samples at room temperature for 1 h. Then, 50 µL of virus-antibody mixtures were added to 293T-ACE2-

© 2023 Bellusci L et al. *JAMA Network Open.*

TMPRSS2 cells [10^4 cells/50 µL; gift from Carol Weiss (1)] in a 96-well plate. The input virus with all SARS-CoV-2 strains was the same (2 x 10^5 relative light units/50 µL/well). After a 3 h incubation, fresh medium was added to the wells. Cells were lysed 24 h later, and luciferase activity was measured using One-Glo luciferase assay system (Promega). The assay of each sample was performed in duplicate, and the 50% neutralization titer was calculated using Prism 9 (GraphPad Software). The limit of detection for the neutralization assay is 1:20. Two independent biological replicate experiments were performed for each sample and variation in PsVNA50 titers was <10% between replicates.

Quantification and statistical analysis

Descriptive statistics were performed to determine the geometric mean titer values and were calculated using GraphPad. All experimental data to compare differences among groups were analyzed using Ordinary one-way ANOVA with Tukey's pairwise multiple comparison test in GraphPad Prism version 9.3.1. To ensure robustness of the results, absolute measurements were log2-transformed before performing the analysis.

Data sharing. All data needed to evaluate the conclusions in the article are present in the manuscript.

Acknowledgments

We thank Basil Golding and Keith Peden at FDA for review of the manuscript. We thank Carol Weiss (FDA) for providing plasmid clones expressing SARS-CoV-2 spike variants.

© 2023 Bellusci L et al. *JAMA Network Open.*

eReferences

1. Neerukonda SN, Vassell R, Herrup R, Liu S, Wang T, Takeda K, et al. Establishment of a well-characterized SARS-CoV-2 lentiviral pseudovirus neutralization assay using 293T cells with stable expression of ACE2 and TMPRSS2. *PLoS One.* 2021;16(3):e0248348.
2. Tang J, Lee Y, Ravichandran S, Grubbs G, Huang C, Stauft CB, et al. Epitope diversity of SARS-CoV-2 hyperimmune intravenous human immunoglobulins and neutralization of variants of concern. *iScience.* 2021:103006.
3. Ravichandran S, Coyle EM, Klenow L, Tang J, Grubbs G, Liu S, et al. Antibody signature induced by SARS-CoV-2 spike protein immunogens in rabbits. *Sci Transl Med.* 2020;12(550):eabc3539.
4. Ravichandran S, Lee Y, Grubbs G, Coyle EM, Klenow L, Akasaka O, et al. Longitudinal antibody repertoire in "mild" versus "severe" COVID-19 patients reveals immune markers associated with disease severity and resolution. *Sci Adv.* 2021;7(10).

© 2023 Bellusci L et al. *JAMA Network Open.*

eTable. SARS-CoV-2 variants mutations introduced in the spike plasmid for production of SARS-CoV-2 pseudovirions for analysis in PsVNA

SARS-CoV-2 variant	Mutations constructed in the spike plasmids
**Omicron (BQ.1)**	BA.4/BA.5 spike mutations (T19I, delL24, delP25, delP26, A27S, del69 70, G142D, V213G, G339D, S371F, S373P, S375F, T376A, D405N, R408S, K417N, N440K, L452R, S477N, T478K, E484A, F486V, Q498R, N501Y, Y505H, D614G, H655Y, N679K, P681H, N764K, D796Y, Q954H, N969K) + K444T and N460K.
**Omicron (BQ.1.1)**	BQ.1 spike mutations (T19I, delL24, delP25, delP26, A27S, del69 70, G142D, V213G, G339D, S371F, S373P, S375F, T376A, D405N, R408S, K417N, N440K, L452R, S477N, T478K, E484A, F486V, Q498R, N501Y, Y505H, D614G, H655Y, N679K, P681H, N764K, D796Y, Q954H, N969K, K444T and N460K) + R346T.
**Omicron (XBB.1)**	BA.2 spike mutations (T19I, delL24, delP25, delP26, A27S, G142D, V213G, G252V, G339D, S371F, S373P, S375F, T376A, D405N, R408S, K417N, N440K, S477N, T478K, E484A, Q493R, Q498R, N501Y, Y505H, D614G, H655Y, N679K, P681H, N764K, D796Y, Q954H, N969K) + V83A, Del144, H146Q, Q183E, V213E, G339H, R346T, L368I, V445P, G446S, N460K, F486S, F490S, R493Q
**Omicron (XBB.1.5)**	BA.2 spike mutations (T19I, delL24, delP25, delP26, A27S, G142D, V213G, G252V, G339D, S371F, S373P, S375F, T376A, D405N, R408S, K417N, N440K, S477N, T478K, E484A, Q493R, Q498R, N501Y, Y505H, D614G, H655Y, N679K, P681H, N764K, D796Y, Q954H, N969K) + V83A, Del144, H146Q, Q183E, V213E, G339H, R346T, L368I, V445P, G446S, N460K, F486P, F490S, R493Q

© 2023 Bellusci L et al. *JAMA Network Open.*
