# Supplementary material for: Comparison of SARS-CoV-2 Hyperimmune Immunoglobulins Following Infection Plus Vaccination vs Infection
Source: JAMA Netw Open. 2023 Aug 4;6(8):e2327307. doi: 10.1001/jamanetworkopen.2023.27307 (PMC10403779; doi:10.1001/jamanetworkopen.2023.27307)
Supplement: Supplement 2. — Data Sharing Statement [file jamanetwopen-e2327307-s002.pdf]

Data Sharing Statement

Bellusci. Comparison of SARS-CoV-2 Hyperimmune Immunoglobulins Following Infection Plus Vaccination vs Infection. *JAMA Netw Open*. Published August 04, 2023.
doi:10.1001/jamanetworkopen.2023.27307

Data
Data available: Yes
Data types: Deidentified participant data
How to access data: Data is provided in the supplementary table S2.
When available: With publication

Supporting Documents
Document types: None

Additional Information
Who can access the data: Anyone requesting the data.
Types of analyses: For any purpose.
Mechanisms of data availability: Data provided in the supplementary table S2.
